# Patient experiences of continuous glucose monitoring and sensor‐augmented insulin pump therapy for diabetes: A systematic review of qualitative studies

**DOI:** 10.1111/1753-0407.13454

**Published:** 2023-08-08

**Authors:** Patrizia Natale, Sharon Chen, Clara K. Chow, Ngai Wah Cheung, David Martinez‐Martin, Corinne Caillaud, Nicole Scholes‐Robertson, Ayano Kelly, Jonathan C. Craig, Giovanni Strippoli, Allison Jaure

**Affiliations:** ^1^ Sydney School of Public Health The University of Sydney Sydney Australia; ^2^ Department of Precision and Regenerative Medicine and Ionian Area (DIMEPRE‐J) University of Bari Aldo Moro Bari Italy; ^3^ Nephrology, Dialysis and Transplantation Unit, Department of Medical and Surgical Sciences University of Foggia Foggia Italy; ^4^ Centre for Kidney Research The Children's Hospital at Westmead Sydney Australia; ^5^ Westmead Applied Research Centre Westmead Hospital Sydney Australia; ^6^ Sydney Medical School The University of Sydney Sydney Australia; ^7^ Westmead Clinical School Westmead Hospital Sydney Australia; ^8^ The University of Sydney Nano Institute (Sydney Nano) The University of Sydney Sydney Australia; ^9^ School of Biomedical Engineering The University of Sydney Sydney Australia; ^10^ Charles Perkins Centre The University of Sydney Sydney Australia; ^11^ School of Medical Sciences The University of Sydney Sydney Australia; ^12^ School of Health and Medicine, South Western Sydney Campus University of New South Wales Sydney Australia; ^13^ Rheumatology Department Liverpool Hospital Sydney Australia; ^14^ Ingham Institute of Applied Medical Research Sydney Australia; ^15^ College of Medicine and Public Health Flinders University Adelaide Australia

**Keywords:** continuous glucose monitoring, insulin pump therapy, patient experiences, type 1 diabetes, type 2 diabetes, 连续血糖监测, 胰岛素泵治疗, 患者体验, 1型糖尿病, 2型糖尿病

## Abstract

**Aims:**

Blood glucose control is central to the management of diabetes, and continuous glucose monitoring (CGM) improves glycemic control. We aimed to describe the perspectives of people with diabetes using CGM.

**Materials and methods:**

We performed a systematic review of qualitative studies.

**Results:**

Fifty‐four studies involving 1845 participants were included. Six themes were identified: gaining control and convenience (reducing pain and time, safeguarding against complications, achieving stricter glucose levels, and sharing responsibility with family); motivating self‐management (fostering ownership, and increasing awareness of glycemic control); providing reassurance and freedom (attaining peace of mind, and restoring social participation); developing confidence (encouraged by the endorsement of others, gaining operational skills, customizing settings for ease of use, and trust in the device); burdened with device complexities (bewildered by unfamiliar technology, reluctant to rely on algorithms, overwhelmed by data, frustrated with malfunctioning and inaccuracy, distressed by alerts, and bulkiness of machines interfering with lifestyle); and excluded by barriers to access (constrained by cost, lack of suppliers).

**Conclusions:**

CGM can improve self‐management and confidence in patients managing diabetes. However, the technical issues, uncertainty in readings, and cost may limit the uptake. Education and training from the health professionals may help to reduce the practical and psychological burden for better patient outcomes.

## Graphical abstract

Highlights

We identified that CGM can increase motivation for self‐management and confidence in treatments. A supportive treating team may help to reduce the practical and psychological burden in patients with type 1 and type 2 diabetes.

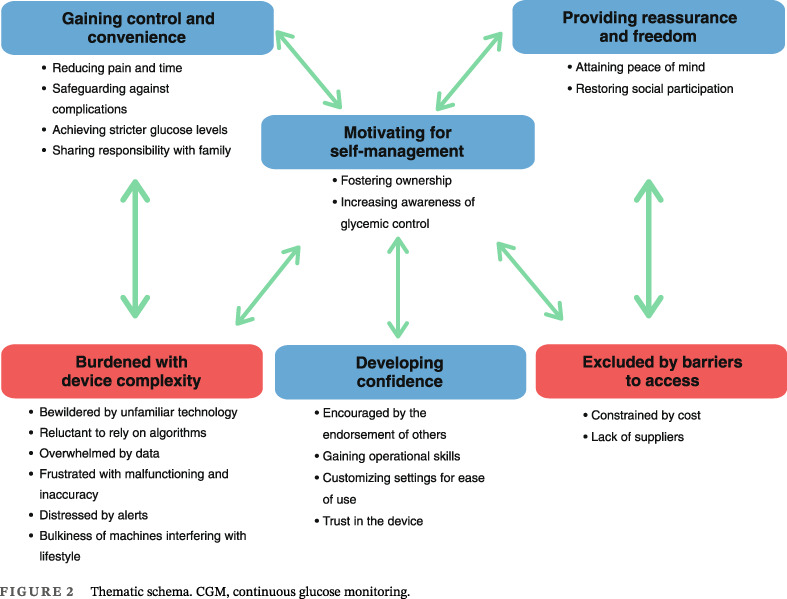

## 摘要

目的:血糖控制是糖尿病管理的核心，连续血糖监测(CGM)可改善血糖控制。我们的目的是描述糖尿病患者使用CGM的观点

方法:我们对定性研究进行了系统综述

结果:纳入了54项研究，涉及1845名参与者。确定了六个主题:获得控制和便利(减少疼痛和时间，防止并发症，实现更严格的血糖控制，与家人分担责任)；激励自我管理(培养主人翁意识，提高血糖控制意识)；提供保证和自由(获得安心，恢复社会参与)；培养信心(受到他人认可的鼓励，获得操作技能，定制设置以方便使用，并对设备充满信任)；设备复杂性带来的负担(被不熟悉的技术所困扰，不愿意依赖算法，被数据压倒，对故障和不准确感到沮丧，被警报困扰，机器笨重干扰了生活方式)；被准入障碍排除(受到成本和缺乏供应商的限制)

结论:CGM可以改善患者糖尿病的自我管理和信心。然而，技术问题、读数的不确定性和成本可能限制其应用。卫生专业人员的教育和培训可能有助于减轻患者的实际和心理负担，以获得更好的患者结果

## 1 INTRODUCTION

Diabetes affects over 422 million people worldwide with rising prevalence.^1^, ^2^ Diabetes is the ninth leading cause of death; it is associated with an increased risk of mortality, cardiovascular events, kidney failure, limb amputation and poor quality of life and imposes a substantial burden on the health system.^1^, ^2^, ^3^, ^4^, ^5^

Blood glucose control is central to the management of diabetes, yet approximately half of people with diabetes do not meet their glucose control targets, an outcome influenced by barriers or challenges with glucose monitoring.^6^, ^7^ About 70% of people with diabetes use standard monitoring of blood glucose; however, repeated finger pricks can be painful and the method does not capture intraday variations.^8^ Continuous glucose monitoring (CGM) has been developed to address these issues. CGM has been shown to improve glycemic control in patients with type 1 and type 2 diabetes, decreasing glycated hemoglobin (HbA1c) and rates of hospitalization for hypoglycemia.^9^, ^10^, ^11^, ^12^, ^13^, ^14^ However, uptake has been very limited and more than 60% of people with diabetes do not use CGM, because of complexity, limited access, and cost due to limited reimbursement from health care to ensure long‐term use.^15^, ^16^ Patients have reported discomfort in the bulkiness of the device, annoyance from the sensor alarm, and irritation or pain in the insertion site.^17^

Little remains known about the patient experiences of CGM for diabetes. A systematic review of qualitative studies can generate comprehensive insight on participant perspectives across different settings and populations. We aim to describe patient expectations and experiences of using CGM and sensor‐augmented insulin pump therapy for type 1 and type 2 diabetes to optimize the acceptability and impact of these devices for better glucose management.

## 2 METHODS

We followed the Enhancing Transparency of Reporting the Synthesis of Qualitative Research (ENTREQ) framework to report our study.^18^

### 2.1 Selection criteria

Qualitative studies and mixed methods studies that reported the perspectives and experiences of adults (≥18 years) with type 1 or type 2 diabetes on CGM (including both flash CGM that transmits the data on demand and real‐time CGM that sends the data instantaneously^19^) without restrictions based on year or study duration. Studies that addressed both automated insulin delivery (AID) and CGM separately were included but only data on CGM were extracted and analyzed in this review. Studies that addressed AID only were excluded. Quantitative studies (eg, randomized controlled trials, cohort studies with no qualitative evaluations), nonprimary research articles, basic science studies, economic studies, quantitative surveys, or studies that reported perspectives from health professionals, caregivers, or people without a diagnosis of diabetes were also excluded. Non‐English articles were excluded to avoid errors that may occur in translation.

### 2.2 Data sources and searches

We searched MEDLINE, Embase, PsycINFO, and CINAHL from inception to 19 April 2023 (Table S1). The search strategy and search terms used are outlined in Table S1. We searched the reference lists of relevant studies and Google Scholar. Three reviewers (PN, SC, AK) independently screened the title and abstracts for inclusion and discarded those that did not meet the inclusion criteria. Full texts were reviewed, and eligible studies were included. Any discrepancies were resolved by discussion with reviewer AJ.

### 2.3 Appraisal of transparency of reporting

The transparency of reporting of each included primary study was assessed using an adapted Consolidated Criteria for Reporting Qualitative Health Research (COREQ)^20^ framework. Three reviewers independently assessed each study and discrepancies were resolved after discussion with another reviewer.

### 2.4 Data analysis

Thematic synthesis was used to inductively identify concepts.^21^ All participant quotations and text from the “results” and “discussion/conclusion” section of each study were also extracted. Two reviewers (PN, SC) coded the data line by line by using HyperRESEARCH software (version 4.5.1) and inductively developed a preliminary coding framework that captured the perspectives of patients with diabetes on CGM and sensor‐augmented insulin pump therapy. We coded the text from each study into these concepts, creating new concepts as needed, and then categorized similar concepts into broader themes. Investigator triangulation was achieved by discussing the preliminary themes with a third reviewer to ensure the findings captured the full range and depth of the data. We developed an analytical thematic schema to represent conceptual patterns and links among the themes.

## 3 RESULTS

### 3.1 Literature search and study description

**FIGURE 1:**
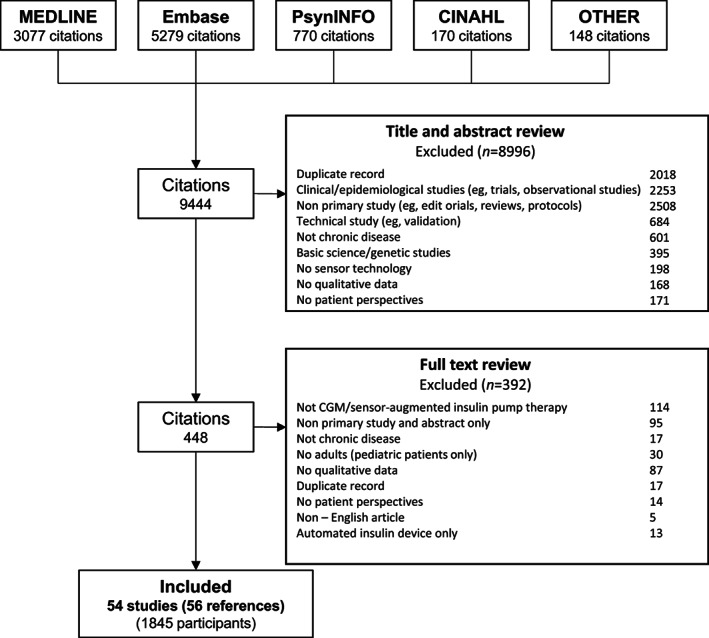
Search results.

We included 54 articles (56 citations) involving 1845 participants (five studies did not report the number of participants) between 18 and 91 years (Figure 1). Of these, eight studies (15%) were performed only in people with type 2 diabetes, three studies (6%) were performed in people with both type 1 and type 2 diabetes, three studies (5%) did not report the type of diabetes, and the remaining studies were conducted in people with type 1 diabetes. The studies were carried out across 18 countries between 2010 and 2023. Twenty‐eight (52%) studies included patient perspectives on CGM with insulin pump therapy, nine (17%) addressed flash CGM, two (4%) used real‐time CGM without providing any information about insulin pump, two (4%) addressed CGM alone without providing any information on the type, one (2%) used both flash and real‐time CGM alone, three (6%) addressed insulin pump alone, and nine (17%) used AID, CGM, and/or insulin pump. Thirty‐three‐ studies (59%) used semistructured or in‐depth interviews, 14 (26%) used focus groups, one study (2%) reported both semistructured interviews or focus groups, four (7%) used open‐ended questions in a questionnaire, and two (4%) were document analyses. The participants and study characteristics of included studies are shown in Table 1.

**TABLE 1:** Characteristics of the included studies.

Study ID	Country	Population (*n*)		Age (years)	*N*	Sex (*n*)		Device	Methodological framework	Data collection	Data analysis	Context/scope
		T1DM	T2DM			M	F					
Addala 2021^22^	US, UK	•		NS	NS	NS	NS	Automated insulin delivery, CGM, insulin pump	Qualitative study	Focus groups	Thematic analysis	Cost
Adu 2019^a^, ^23^	Multinational	•	•	Mean 44.6	217^a^	94	123	CGM, insulin pump	Qualitative study	Semistructured interviews	Thematic analysis	Self‐management
Agarwal 2021^24^	US	•		Mean 21.5	40	24	16	CGM, insulin pump	Qualitative study	Semistructured interviews	Thematic analysis	Patient perception
Allen 2021^25^	US	•		Mean 44	134	53	81	CGM, insulin pump	Qualitative study	Semistructured interviews	Thematic analysis	Expectations and experiences
Barnard 2017^26^	UK, Austria	•		Mean 38.6	32	18	14	Closed‐loop (Dana Diabecare R), CGM (FreeStyle Navigator II), sensor augmented insulin pump therapy (open loop)	Qualitative study	Semistructured interviews	Thematic analysis	Expectations and experiences
Bispham 2021^27^	US	•		Mean 44.3	17	NS	NS	CGM, insulin pump	Qualitative study	Focus groups	Thematic analysis	Expectations and experiences
Boucher 2019^28^	New Zealand	•		18–19	7	5	2	fCGM (FreeStyle Libre)	Qualitative study	Semistructured interviews	Thematic analysis	User experience
Burniside 2023^29^	New Zealand	•		NS	6	NS	NS	Open‐source AID (OpenAPS algorithm in a modified version of the Android application), sensor‐augmented pump therapy (DANA‐i insulin pump, and Dexcom G6 CGM)	Qualitative study	Semistructured interviews	Thematic analysis	User experience
Chang 2017^30^	Taiwan		•	65–91	18	6	12	Flash glucose	Qualitative study	Semistructured interviews	Thematic analysis	User experience
Chesser 2022^31^	US		•	NS	NS	NS	NS	CGM (Dexcom G6)	Mixed modality	Focus groups	Thematic analysis	Expectations and experiences
Chiu 2019^32^	Taiwan		•	53–72	20	13	7	CGM (iPro 2), insulin pump	Qualitative study	Semistructured interviews	Thematic analysis	User experience
Cleal 2021^33^	Denmark	•		Mean 48	21	11	10	isCGM, CGM, insulin pump	Qualitative study	Semistructured interviews	Thematic analysis	User experience
Cuevas 2022^34^	US		•	Mean 68.5	30	16	14	CGM	Mixed modality	Semistructured interviews	Thematic analysis	User experience
Dutil 2014^35^	Canada	•		18–64	6	2	4	CGM, insulin pump (Tandem pump)	Qualitative study	Semistructured interviews	Thematic analysis	Patient perception
Farrington 2018^36^	UK	•		18–45	16	0	16	Closed‐loop (Florence D2a), CGM (FreeStyle Navigator 2), insulin pump (DANA Diabecare R Insulin Pump SOOIL)	Qualitative study	Semistructured interviews	Thematic analysis	User experience
Faulds 2022^c^, ^37^	US	•		NS	12	NS	NS	Automated insulin delivery, CGM	Mixed modality	Focus groups	Thematic analysis	Expectations and experiences
Fritschi 2022^38^	US		•	Mean 68	8	0	8	fCGM (FreeStyle Libre) and Fitbit	Qualitative study	Semistructured interviews	Thematic analysis	Self‐management
Gajewska 2021^39^	Ireland	•		NS	28	NS	NS	Continuous subcutaneous insulin infusion, CGM (Libre), insulin pump	Qualitative study	Semistructured focus groups	Thematic analysis	User experience
Grando 2019^40^	US	•		NS	4	NS	NS	Closed‐loop insulin pump (Medtronic MiniMed 670G), CGM (Enlite, Dexcom)	NA	Questionnaire with open text responses	Thematic analysis	Experience and satisfaction
Griauzde 2022^41^	US		•	NS	21	NS	NS	CGM (Freestyle Libre)	Mixed modality	Semistructured interviews	Thematic analysis	User experience
Grigorian 2022^42^	US	•		NS	NS	NS	NS	CGM, insulin pump	Qualitative study	Semistructured interviews	Thematic analysis	Self‐management
Haynes 2021^43^	Canada	•		18–25	6	NS	NS	CGM, insulin pump	Qualitative study	Semistructured interviews	Thematic analysis	User experience
Hendrieckx 2017^44^	Australia	•		Mean 42	16	7	9	Closed‐loop, CGM, insulin pump	Qualitative study	Semistructured interviews	Descriptive	User experience
Hughes 2022^45^	US	•		Mean 49	38	19	19	CGM	Phenomenological qualitative approach	Focus groups	Thematic analysis	Expectations and experiences
Kahkoska 2023^46^, ^47^	US	•		NS	22	NS	NS	CGM (Dexcom, Freestyle Libre, Medtronic), insulin pump, hybrid closed loop system	Qualitative study	Focus groups	Thematic analysis	Expectations and experiences
Kang 2022^48^	Korea	•	•	Mean 44.5	19	7	12	CGM (FreeStyle Libre)	Qualitative study	Semistructured interviews	Thematic analysis	User experience
Kaisen 2020^49^	US	NS	NS	Mean 49.7	18	NS	NS	Insulin pump	Qualitative study	Semistructured interviews	Thematic analysis	Use in hospital
Kropff 2017^50^	Italy, The Netherlands	•		Mean 47	32	14	18	Closed‐loop, CGM, insulin pump	Qualitative study	Semistructured interviews	Thematic analysis	Satisfaction
Litchman 2018^51^	US	•		NS	NS	NS	NS	rtCGM (Dexcom, MiniMed)	Qualitative study	Document analysis	Thematic analysis	User experience and data share
Litchman 2021^52^, ^53^	US		•	Mean 54.8	26	10	16	CGM (FreeStyle Libre) + online peer support community	Qualitative study	Semistructured interviews	Thematic analysis	User experience
Lukacs 2022^54^	Hungary	•		NS	94	NS	NS	CGM (Medtronic, Enlite, Guardian 3, Abbott Freestyle Libre), insulin pump	NA	Questionnaire with open text responses	Thematic analysis	Experience and satisfaction
Nadeem 2021^b^, ^55^	Pakistan	•	•	NS	37	NS	NS	CGM, insulin pump, continuous subcutaneous insulin infusion	NA	Questionnaire with open text responses	Descriptive	Patient perception
Overend 2019^56^	UK	•		20–79	40	18	22	fCGM (FreeStyle Libre)	Qualitative study	Semistructured interviews	Thematic analysis	Impact on quality of life
Persson 2022^57^	Sweden	•		Mean 51.6	37	14	23	CSII, CGM	Qualitative study	Focus groups	Thematic analysis	Expectations and experiences
Pickup 2015^58^	UK	•		Mean 44.4	50	23	27	CGM (Medtronic Veo/Enlite, Medtronic Guardian Real‐Time, Medtronic 522, Medtronic 722, Medtronic Veo/Soft‐Sensor, Dexcom G4 Platinum, Dexcom Seven Plus, Animas Vibe), insulin pump, CSII	NA	Questionnaire with open text responses	Framework analysis	User experience
Pillalamarri 2018^59^	US	NS	NS	NS	>343	NS	NS	Bluetooth enabled tubeless insulin pump (Omnipod DASH)	NA	In‐home ethnographic visits, interviews	NS	User experience
Ritholz 2019^60^	US	•		Mean 52	10	6	4	CGM (FreeStyle Libre), insulin pump	Qualitative study	Semistructured interviews	Thematic analysis	User experience
Ritholz 2014^61^	US	•		30–70	20	10	10	CGM, insulin pump	Qualitative study	Focus groups	Thematic analysis	Expectations and experiences
Ritholz 2010^62^	US	•		Mean 45	20	10	10	CGM, insulin pump	Qualitative study	Semistructured interviews	Thematic analysis	Psychosocial experiences
Sawyer 2021^63^	US	•		18–30	21	2	19	CGM, insulin pump	Qualitative study	Semistructured interviews	Thematic analysis	Patient perception
Shephard 2012^64^	US	•		Mean 41	56	23	33	CGM (DexCom 7 Plus), insulin pump (Omnipod)	Qualitative study	Focus groups	Thematic analysis	Attitude and concerns
Sørgård 2019^65^	Norway	•		21–68	23	11	12	CGM, insulin pump	Qualitative study	Semistructured interviews	Thematic analysis	User experience
Stuckey 2021^66^	US	•		NS	NS	NS	NS	CGM, insulin pump	Qualitative study	Internet blogs	Thematic analysis	User experience
Tanenbaum 2021a^67^	US	•		Mean 31	22	9	13	CGM (Dexcom, FreeStyle Libre, Medtronic Guardian sensor), insulin pump	Qualitative study	Semistructured focus groups	Thematic analysis	Patient perception
Tanenbau 2021b^68^	US	•		Mean 31	22	9	13	CGM (Dexcom, Medtronic, Abbott), insulin pump (Medtronic, Tandem, Insulet) + telehealth	Qualitative study	Semistructured focus groups	Thematic analysis	User experience on telehealth
Toschi 2022^69^	US	•		Mean 71.0	34	16	18	CGM, insulin pump	Mixed modality	Semistructured interviews	Thematic analysis	User experience on telehealth during the COVID‐19 lockdown
Tzivian 2022^70^	Latvia	•		18–50	21	6	15	Insulin pump	Mixed modality	Semistructured interviews	Thematic analysis	Expectations and experiences
Vallis 2022^71^	Canada		•	25–65	13	2	11	CGM (FreeStyle Libre, FreeStyle Libre 2, Dexcom)	Qualitative study	Semistructured interviews	Thematic analysis	User experience
Van Heerden 2022^72^	South Africa	NS	NS	Mean 29.8	10	0	10	CGM (Freestyle Libre 2)	Mixed modality	Semistructured interviews	Thematic analysis	Expectations and experiences
Vlcek 2023^73^	Canada	•		19–62	22	8	14	CGM, insulin pump	Qualitative study	Semistructured interviews or focus group	Thematic analysis	Expectations and experiences
Vloemans 2017^74^	Netherlands	•		Mean 47.7	23	13	10	CGM, insulin pump, CSII	Qualitative study	Semistructured interviews	Thematic analysis	User experience
Walker 2021^75^	US	•		Mean 42	86	NS	NS	CGM, insulin pump	Qualitative study	Semistructured focus groups	Thematic analysis	Patient perception
Wallace 2023^76^	UK	•		24–66	15	5	10	fCGM	Qualitative study	Semistructured interviews	Thematic analysis	User experience
Yorgason 2021^77^	US	•		Mean 24	12	5	7	CGM, insulin pump	Qualitative study	Focus groups	Thematic analysis	Patient perception

*Note*: Dot denotes the population reported in the study.

^a^ Adu 2019 included participants with type 1 diabetes mellitus (*n* = 83) and type 2 diabetes (*n* = 134).

^b^ Nadeem 2021 included participants with type 1 diabetes mellitus and type 2 diabetes, but numbers were not clearly reported.

^c^ Data only of CGM users were included.

Abbreviations: AID, automated insulin delivery; CGM, continuous glucose monitoring; CSII, continuous subcutaneous insulin infusion; F, female; fCGM, flash continuous glucose monitoring; isCGM, intermittently scanned continuous glucose monitoring; M, male; NS, not stated; rtCGM, real‐time continuous glucose monitoring; UK, United Kingdom; US, United States.

### 3.2 Comprehensiveness of reporting

The comprehensiveness of reporting among the included articles is shown in Table 2. Of the 26 possible items included in the adapted COREQ framework, studies reported between 6 and 23 of these. The participant selection strategy and participant characteristics were reported in 30 (56%) and 45 (83%) studies respectively. Twenty‐five (46%) studies specified theoretical or data saturation, and 38 (70%) studies described researcher triangulation. There were eight studies that were not appropriate for the adoption of COREQ framework as they did not obtain their data via interviews or focus groups (Table S2).

**TABLE 2:** Comprehensiveness of reporting in included studies^a^

Item	Studies reporting each item	Number of studies
Personal characteristics		
Interviewer/facilitator identified	(23, 25, 26, 27, 28, 29, 31, 34, 37, 38, 39, 41, 42, 43, 44, 45, 49, 56, 57, 60, 62, 63, 64, 65, 67, 71, 72, 73, 74, 76, 77)	31
Occupation of the interview/facilitator	(23, 25, 27, 28, 29, 34, 38, 41, 42, 43, 44, 45, 46, 49, 56, 57, 60, 62, 63, 65, 67, 71, 72, 73, 74, 75, 76, 77)	28
Experience or training in qualitative research	(23, 25, 28, 29, 30, 32, 38, 41, 42, 43, 44, 49, 60, 61, 67, 71, 72, 73, 75, 77)	20
Relationship with participants		
Relationship established before study start	(23, 25, 26, 28, 29, 38, 41, 42, 44, 48, 52, 56, 57, 63, 65, 67, 68, 70, 71, 76)	20
Participant selection		
Selection strategy	(23, 26, 28, 30, 31, 32, 33, 34, 36, 37, 38, 39, 41, 43, 46, 48, 52, 56, 60, 61, 62, 63, 65, 67, 68, 71, 72, 73, 74, 77)	30
Method of approach or recruitment	(23, 26, 27, 28, 29, 31, 34, 35, 38, 39, 41, 42, 43, 45, 46, 48, 49, 52, 56, 57, 60, 61, 62, 63, 64, 65, 67, 68, 70, 71, 73, 75, 76)	33
Sample size	(22, 23, 24, 25, 26, 27, 28, 29, 30, 32, 33, 34, 35, 36, 37, 38, 39, 41, 42, 43, 44, 45, 46, 48, 49, 50, 52, 56, 57, 60, 61, 62, 63, 64, 65, 67, 68, 69, 70, 71, 72, 73, 74, 75, 76, 77)	46
No. and/or reasons for nonparticipation	(23, 24, 28, 29, 31, 36, 41, 43, 52, 57, 60, 61, 62, 64, 65, 68, 70, 71, 74, 76)	20
Setting		
Venue of data collection	(23, 24, 27, 28, 29, 30, 31, 32, 33, 34, 37, 38, 41, 42, 43, 44, 45, 46, 48, 49, 52, 56, 57, 60, 63, 65, 67, 69, 70, 71, 72, 73, 76)	33
Presence of nonparticipants (eg, clinical staff)	(23, 29, 46, 75)	4
Description of sample	(22, 23, 24, 25, 26, 27, 28, 29, 30, 31, 32, 33, 34, 35, 36, 37, 38, 39, 41, 42, 43, 44, 45, 46, 48, 49, 56, 57, 60, 61, 62, 63, 64, 65, 67, 68, 69, 70, 71, 72, 73, 74, 75, 76, 77)	45
Data collection		
Questions, prompts or topic guide	(23, 24, 25, 26, 27, 28, 29, 30, 31, 32, 33, 34, 35, 36, 38, 39, 42, 43, 44, 45, 46, 48, 49, 50, 56, 60, 61, 62, 63, 64, 65, 67, 69, 71, 72, 73, 74, 75, 76, 77)	40
Repeat interviews/observations	23, 26, 32, 36, 42, 43, 56, 65	8
Audio/visual recording	(23, 24, 25, 26, 27, 28, 29, 30, 32, 34, 35, 36, 37, 38, 39, 41, 42, 43, 44, 45, 46, 48, 49, 52, 57, 60, 61, 62, 63, 65, 67, 68, 69, 70, 71, 72, 73, 74, 75, 76, 77)	41
Field notes	(23, 24, 30, 35, 37, 45, 49, 61, 63, 65, 71, 75, 77)	13
Duration of data collection	(22, 23, 26, 28, 29, 30, 31, 32, 33, 34, 38, 39, 42, 43, 44, 45, 46, 48, 57, 60, 61, 62, 63, 64, 65, 67, 69, 71, 72, 74, 75, 76, 77)	33
Translation and interpretation	(50, 52, 70, 72, 73, 75)	6
Protocol for data preparation and transcription	(23, 24, 25, 26, 27, 28, 29, 30, 32, 33, 35, 36, 43, 44, 45, 48, 49, 57, 60, 61, 62, 63, 64, 65, 73, 74, 75, 76)	30
Data (or theoretical) saturation	(22, 23, 24, 26, 28, 29, 32, 35, 36, 39, 41, 43, 46, 48, 49, 50, 56, 60, 61, 62, 67, 70, 71, 72, 73, 75, 77)	25
Data analysis		
Researcher/expert triangulation	(22, 23, 24, 25, 26, 27, 28, 29, 30, 31, 34, 35, 37, 38, 39, 41, 42, 43, 44, 45, 46, 48, 49, 56, 57, 60, 61, 62, 65, 67, 68, 69, 71, 73, 74, 75, 76, 77)	38
Translation	(50, 52, 65, 70, 72, 73)	6
Derivation of themes or findings	(22, 23, 24, 25, 26, 27, 28, 29, 30, 31, 32, 33, 34, 35, 36, 37, 38, 39, 41, 42, 43, 44, 45, 48, 49, 52, 56, 57, 60, 61, 62, 63, 64, 65, 67, 68, 69, 70, 71, 72, 73, 74, 75, 76, 77)	45
Use of software	(22, 23, 24, 25, 28, 29, 30, 33, 34, 36, 37, 39, 41, 42, 43, 44, 46, 48, 57, 60, 61, 65, 67, 69, 70, 71, 72, 73, 76)	29
Member checking	(29, 31, 38, 41, 42, 43, 46, 57, 62, 65, 67, 71, 73, 76, 77)	15
Reporting		
Participant quotations or raw data provided	(22, 23, 24, 25, 26, 27, 28, 29, 30, 31, 32, 33, 34, 35, 36, 38, 39, 41, 42, 43, 44, 45, 46, 48, 49, 50, 56, 57, 60, 61, 62, 63, 65, 67, 68, 69, 70, 71, 72, 73, 74, 75, 76, 77)	44
Range of depth of insight into participant perspectives on CGM	(23, 24, 25, 28, 30, 32, 33, 34, 35, 38, 39, 43, 46, 48, 49, 56, 57, 60, 61, 62, 63, 65, 67, 69, 71, 72, 74, 75, 76, 77)	30

Abbreviations: CGM, continuous glucose monitoring; COREQ, Consolidated Criteria for Reporting Qualitative Health Research.

^a^ Only interview and focus group studies were included in the COREQ assessment.

### 3.3 Synthesis

**FIGURE 2:**
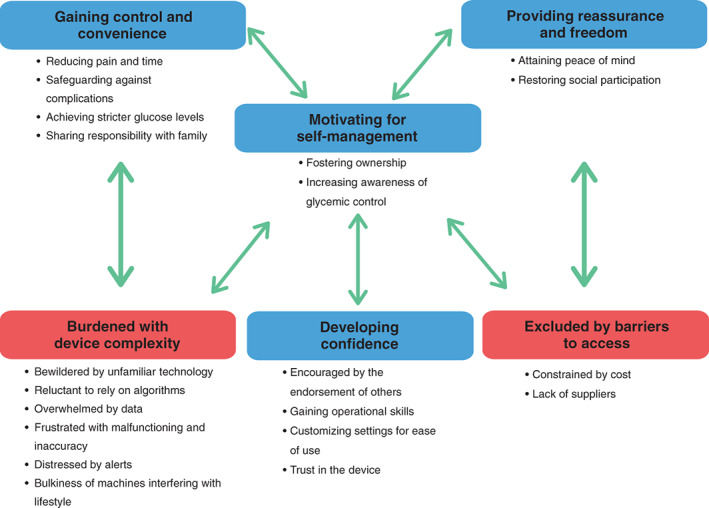
Thematic schema. CGM, continuous glucose monitoring.

We identified six themes: gaining control and convenience, motivating self‐management, providing reassurance and freedom, developing confidence and trust, burdened with device complexities, and excluded by barriers to access. Selected illustrative participant quotations for each theme are provided in Table 3. The conceptual relationship among the themes is depicted in Figure 2.

**TABLE 3:** Selected quotations from primary studies to illustrate each theme.

Theme	Quotations	Sources
Gaining control and convenience		
Reducing pain and time	“Taking away finger‐pricks makes a big difference”, “I was sick to death of blood glucose monitoring.”^56^ “The challenge for me, before I started on the sensor, was that I do not take the time… to test; if it is unpractical for me in a busy work schedule, or a regular day or whatever, it was not something I prioritized.”^65^ “[CGM] was a lot better. I did not have sore fingers anymore…I was always embarrassed to find strips in my car and strips on the floor at home. But here I was thinking I was so good at getting rid of them.”^71^	(23, 28, 34, 41, 46, 48, 52, 55, 56, 58, 60, 65, 69, 71)
Safeguarding against complications	“I have not had a severe hypo for 4 months and only one when I needed medical help in 18 months of usage, previously I was in hospital 2 to 3 times a month.”^58^ “It's one thing to hear ‘avoid being low it could kill you,’ which had not been my experience with the first 40–50 years of management, but seeing it happen and nipping it in the bud, I could see a downward trend or a fast downward trend, I could catch it before it became an issue, and the same on the high ends. Yeah it was definitely good to avoid the extremes.”^60^ “It's because I've got the sensor and the pump. So, I've actually got the chance to do something. Whereas before, when I just pricked my finger, I could not do anything. Because my blood sugar could go up like a mountain before I even noticed that things were moving in the wrong direction. So, when you make that one‐off measurement it might well be fine.”^33^	(25, 28, 33, 41, 43, 45, 46, 51, 54, 55, 56, 58, 60, 61, 62, 65, 69, 71, 74)
Achieving stricter glucose levels	“My new mantra is live your life as if you did not have diabetes. […] It was the ability to live as I do not have diabetes. It means freedom and keeping A1c below 7, a healthy A1C really, and minimizing fear of long‐term complications.”^35^ “I tried to maintain my glucose levels within the normal range. I realized that checking glucose levels is essential, and I exercised more in the morning or reduced the amount of food if my fasting glucose levels rose above the normal limits.”^48^ “Now with the sensor […] my A1Cs have been almost on the dot comparatively to my blood test at the hospital.”^71^	(28, 32, 35, 38, 48, 56, 58, 60, 65, 71, 74)
Sharing responsibility with family	“He had tears in his eyes telling me what it means to him that he can now keep me safer. I did not set the share alarm to alert him if my blood sugar dropped too low, not wanting to unnecessarily burden him. He set it for himself. He said, ‘I can better protect you now.’ And I realize I do feel safer.”^51^ “Both the pump and continuous glucose monitoring have been a godsend for us. Initially, I looked at it more as it was good for her, but it's for me because I can wake up in the morning and if I do not hear that thing beeping or if I wasn't woken up in the middle of the night, I can let her sleep another hour… It was nice for me to have some way of knowing what was going on…”^61^	(25, 28, 35, 51, 56, 59, 61, 62, 65, 73, 74, 76)
Motivating self‐management		
Fostering ownership	“I can only be positive and in two sentences: I was in the middle of a diabetes‐burnout, and I am now sure of myself and that is what it brought me. This study I think is called ‘IN CONTROL’, well that's it.”^74^ “All I can answer is that I am doing this [experiment] for my own good! And I also want to know where the problem lies!”^32^ “For long‐term monitoring and understanding it makes it so much better and easier, and you pay more attention to your diabetes.”^56^	(23, 28, 30, 32, 34, 35, 38, 41, 43, 48, 49, 51, 56, 58, 60, 61, 65, 71, 74, 76)
Increasing awareness of glycemic control	“When I have this arrow, a number, and the trend information, I think ahead much more. I can see in which direction I am headed and think through how much insulin I use and what I eat. I am much more on top of things.”^65^ “‘Blood glucose is just a snapshot – the trends are really useful’, ‘The arrows help me predict and plan.’”^56^ “…to try and be an active learner and pay attention to what's happening.”^23^ “Well just seeing where my blood sugars were going, and being able to keep track of everything in one location, what I was eating, my activity level, um, my insulin dosages, and then being able to see snapshots of where you went low when you went for a 2 mile walk and just compare it to a day where I sat at my desk all day It really helped me to understand how to better adjust my insulin dosages, to better reflect, or to have better control and fewer fluctuations.”^60^	(23, 24, 25, 28, 30, 31, 32, 33, 34, 35, 38, 41, 43, 52, 54, 56, 58, 60, 61, 62, 63, 65, 68, 70, 71, 73, 74, 76)
Providing reassurance and freedom		
Attaining peace of mind	“I think it [Sugar Sleuth] just made it [diabetes] um, easier to manage; easier is not really the right word, but it just, it just, it enhanced having it [diabetes]. I did not think about it more. I did not think about it less; it just made dealing with it [diabetes] more pleasant.”^60^ “CGM has changed my life. Prior to starting it I could not be left on my own for fear of an unpredictable hypo. Since starting it, my life has changed totally for the better.”^58^ “I slept better with my [RT‐CGM] rigged up. [My husband] could see my data while I was sleeping, and his system would alert him to any overnight hypos, should they occur. That's some good peace of mind for me when I'm a plane ride away from my support system.”^51^	(25, 33, 34, 38, 48, 49, 51, 54, 55, 57, 58, 60, 69, 71, 72, 74, 76)
Restoring social participation	“One of the advantages is managing my BG during exercised I am able to monitor while at the gym without finger sticks every 5 minutes and can come out of the gym with virtually the same BG as when I went in.”^58^ “If I go out to play football with my friends, I do not have to be the guy that has to leave early, because (I scan when feeling low) and get something to eat so I can just keep playing.”^28^ “Also, good protection when driving, as I do not always have hypo warning signs. Much easier to work effectively, as no (longer) need to keep stopping and checking BG (blood glucose) via blood tests.”^58^	(28, 38, 43, 48, 51, 54, 56, 57, 58, 65, 69, 76)
Developing confidence		
Encouraged by the endorsement of others	“I use this machine because my attending physician introduced it to me.”^30^ “He [spouse] actually expresses audible excitement when he sees me using it. He's incredibly supportive. He just tried the sensor for 5 days to know what I was going through. He's a big advocate of it, which makes everything easier for me.”^62^ “I had told my mom about it a little bit and she […] had no idea what diabetes was when I was diagnosed. […] She was kind of backing me up while I did all my research and everything.”^35^	(23, 24, 25, 28, 30, 32, 33, 35, 39, 48, 49, 51, 55, 57, 58, 60, 61, 62, 65, 66, 67, 68, 71, 75, 77)
Gaining operational skills	“The nurse told me that I can phone them whenever I have problems with the equipment. I do not like contacting them through phone because I feel that I cannot speak or hear clearly when using a phone; currently, I do not phone them. Instead, I bring the equipment with me during my next check‐up. This allows the nurse to demonstrate the equipment to me in person; this way is more informative.”^30^ “Arrows pointing vertically upward indicate an increase in speed. If the arrows point vertically downward, glucose levels drop within 30 minutes, causing hypoglycemia. It is possible to be prepared.”^48^ “I have different strategies [to use the device]. There are five different programs (in my device), so I can change. For example, if I am ill, I have one (program) with a higher base level of insulin as I need that then. Or for extreme exercise, for example, when I dance all day, then I use another (program) and then I have one (program) that I usually use.”^57^	(28, 30, 33, 35, 46, 48, 57, 59, 60, 62, 65, 67, 68, 77)
Customizing settings for ease of use	“The screen is more aesthetically pleasing compared to the older Medtronic pumps.”^40^ “The teaching for diabetes (insulin, pump, monitor etc.) is pretty simplistic.”^63^ “When I got the Dexcom [CGM}, it was by myself, and it was super easy. I got it going by myself online.”^67^	(30, 35, 40, 51, 57, 59, 60, 61, 63, 65, 67, 74)
Trust in the device	“I am confident in what my Dexcom [CGM] says.”^63^ “10% of the time it's wrong or not reading correctly. I'm clearly going to keep wearing it and trusting it for the other 90% of the time.”^68^	(46, 54, 63, 64, 65, 68, 74)
Burdened with device complexities		
Bewildered by unfamiliar technology	“My wife… knows how to measure blood glucose. I never operate the equipment myself… I cannot remember (how to use the equipment) — I do not want to break it. It (the equipment) is very expensive, I do not have to learn! Too troublesome!”^30^ “Who really uses this [technology] in the overall general public? If there was somebody that's a severe diabetic and is in a low‐income environment… they would not know what this is… I am also talking about other religions, cultures and stuff like that Hispanics, Blacks… they would not understand what this is…”^60^ “I think it could be scary to push a button and… I did not even realize [the sensor] was, like, in you.”^67^	(24, 30, 35, 40, 55, 57, 58, 60, 62, 65, 67, 69)
Reluctant to rely on algorithms	“Sometimes, I can be lazy on the pump. I know that sounds bad! But, if I was on injections I might not be as lazy.”^35^ “Providing data about me is a disturbing feeling.”^54^	(32, 33, 35, 54, 57, 58, 62, 63, 64, 65, 74, 75, 76)
Overwhelmed by data	“It's a lot of information. It's very… it's really useful for me. And then sometimes it's not. […] Sometimes I have a hate relationship with my iPhone because it's really unhealthy, like it's too much information and it's kind of the same with the pump.”^35^ “You are under constant surveillance. And then the arrows go upwards and then they go downwards, and then the alarm sounds if it drops too fast, and if it (blood glucose) rise too quickly you become stressed.”^65^ “In real life, I'm probably not gonna get up every morning and look all night long and say, “oh look I went up, I went down…” It's just not gonna happen… Maybe I do not get the use of all the data but just that arrow going up and down and telling you what's happening right now is enough to make you do stuff.”^60^	(32, 33, 35, 40, 48, 51, 54, 58, 60, 62, 65, 67, 74, 76)
Frustrated with malfunctioning and inaccuracy	“And I did not think it was quite working properly. Sometimes my glucose meter would be way different from the CGM so that kind of thing. The calibration… I hated it.”^35^ “The sensor is very high maintenance. It asks for blood glucose readings constantly. It does not give any data if you do not calibrate every time it asks.”^40^ “When you have something that should help you, and should work, but you end up having a lot of problems, then it becomes so negative, and that I could not stand to use it (CGM), and I just put it away.”^65^	(28, 35, 40, 46, 48, 51, 54, 56, 57, 58, 65, 66, 67, 71, 74, 76)
Distressed by alerts	“What was very frustrating for me and actually made the whole experience less useful was the frequency of the alarms… and the number of beeps… I was ready to hand the whole kit back.”^62^ “Like if you have alarms going off and you are used to shutting them off because they aren't meaningful what happens when an alarm goes off and it is meaningful… they aren't differentiating between the important ones and the ones that are “oh gee whiz is not that great technology… So all of these alarm capabilities these things have, to a great extent, conditions people to ignore alarms.”^35^	(23, 24, 25, 32, 33, 35, 40, 46, 51, 58, 59, 60, 61, 62, 63, 64, 65, 66, 74)
Bulkiness of machines interfering with lifestyle	“Because I had an operation on my waist before, I can easily get a backache. I used to have a hot bubble bath in the morning, but I cannot because I am wearing the device. It is inconvenient now that my activities have become less smooth in the morning.”^32^ “Physically he [spouse] found it odd to be looking at it on my body, especially since I had the pump on one side and the monitor on the other. It was a lot of paraphernalia. I called myself the Bionic Woman at that point… I would want the lights out, a little more often.”^62^ “What if someone did not understand that I had diabetes? What if someone did not know what I was doing? It made me feel like this [giving injections] wasn't something that was appropriate to be doing in a public place.”^43^	(24, 25, 28, 32, 33, 34, 35, 39, 41, 43, 48, 52, 54, 56, 57, 62, 63, 65, 66, 67, 71, 72, 73, 74, 75, 76, 77)
Excluded by barriers to access		
Constrained by cost	“I manage my diabetes fairly closely and I pay for HbA1c, you know… the financial cost is quite large. In Australia, our health system's pretty good but you still have to pay for a lot of equipment which the government does not seem to agree necessarily. Continuous Glucose Monitor should be government funded for over 21 s for Christ sake.”^23^ “I love CGM, but I am frustrated that the NHS will not fund it for me. I worry that I may have to stop using my CGM when finances get tight.”^58^ “Medicare does not even cover CGMs. Unless there's an overwhelming pivotal study that shows that the outcomes are so much better, they are going to try to avoid it at every turn…”^22^	(22, 23, 24, 30, 35, 41, 42, 43, 48, 52, 55, 58, 63, 65, 70, 71, 73)
Lack of suppliers	“… when I was out of sensors, it could easily take 4 days before I got to the hospital to get new ones. So it would have been easier if I could pick up the sensors at the pharmacy together with my insulin.”^65^ “I've come into an issue with insurance, and I do not know how to get a pump. I've been trying to get a pump and a CGM for like 4 years.”^75^ “[There] is not equity for patients because you can do some and you cannot do others. I think you need the resources allocated from the get go (HCP6, high uptake)— Say somebody comes in thinking about a pump, we have developed a resource, it is an information sheet about pump therapy, so some of the pros and cons and then the options that are available in Ireland right now with the websites for the companies.”^39^	(22, 39, 42, 55, 65, 69, 75)

Abbreviations: CGM, continuous glucose monitoring; NHS, National Health Service; RT‐CGM, real‐time continuous glucose monitoring.

## 4 GAINING CONTROL AND CONVENIENCE

### 4.1 Reducing pain and time

CGM was regarded by patients both as less painful and time consuming than the standard monitoring of blood glucose. Participants described the finger prick test as a deterrent to checking blood sugar level, but with CGM they “*no longer [had] sore fingers*.”^56^ Standard monitoring of blood glucose was time consuming to use and patients often had to interrupt their daily activities to perform the test, whereas CGM offered a pain‐free and “*convenient*”^48^ alternative and glucose results that they “*can check instantly*.”^56^

### 4.2 Safeguarding against complications

Participants felt safe in being able to review their glucose level in real time and “*liked [to be] warned when blood sugar was too high or too low*.”^74^ Participants felt empowered to prevent a life‐threatening crisis with alarms to treat their blood glucose promptly. The forecast of glucose trends allowed them to sustain more stable glucose control with less fluctuations. Patients with Type 1 diabetes believed that, using CGM, hypoglycemia was predicted or rapidly detected, and this was especially lifesaving in patients with poor hypoglycemic awareness who enjoyed “*no severe hypos since using*.”^56^

### 4.3 Achieving stricter glucose levels

Participants who used CGM or sensor‐augmented insulin pump therapy felt pleased with the direction of HbA1c. Some mentioned that the artificial pancreas accomplished better glycemic control than they could, and CGM helped them to “*achieve better control than with [standard monitoring of blood glucose]*.”^58^ Patients with type 1 diabetes were motivated to set achievable goals for themselves such as “*to get [HbA1c] below 8*.”^28^

### 4.4 Sharing responsibility with family

Some reported benefit from data sharing as the family and friends could empathize with the patient to improve the control of the disease. Participants felt less lonely and “*exponentially safer*”^61^ knowing there was another person looking out for their blood sugar levels. The collaborative process of managing one's diabetes as a family “*brought [them] closer together because [they were] sharing the experience of diabetes*.”^61^

## 5 MOTIVATING SELF‐MANAGEMENT

### 5.1 Fostering ownership

Gaining more understanding of the management of diabetes empowered patients using CGM alone to take ownership of their treatment—“*[You] need to be monitoring what you eat, and you need to know what your numbers are throughout the day*.”^38^ CGM provided “*a privilege of being in control*”^49^ of diabetes where participants had more “*confidence*”^76^ in their ability to manage their diabetes. Patients with type 2 diabetes enjoyed being able to “*look at the CGM more to see where [the] sugar is going*”^34^ having a “*bigger picture on what was going on with [the] diabetes*”^71^ and subsequently they can “*take their disease more seriously*.”^71^

### 5.2 Increasing awareness of glycemic control

With CGM, participants gained better recognition of their blood glucose trends and an “*understanding how [their] body reacts*”^23^ to their lifestyle choices. The CGM readings encouraged them to “*engage with [their] diabetes control*”^62^ as the trends “*help[ed] [them] predict*”^56^ the fluctuations in glucose levels. They were encouraged to change their dietary and exercise behaviors as they could see stability in their glucose levels on their device and were reassured that they were “*much more on top of things*.”^65^

## 6 PROVIDING REASSURANCE AND FREEDOM

### 6.1 Attaining peace of mind

For participants, the automation of technology and its safety features meant the “*the pump provided a feeling that something of that previous life was recovered*.”^33^ Participants explained that CGM took over the control of diabetes and the responsibilities that came with glucose management. Some felt protected from complications and worried less because it “*felt like [they] had a buddy who watched over [them]*.”^74^

### 6.2 Restoring social participation

Participants were more confident in their glycemic control with CGM, and this enabled them to have a more flexible lifestyle and “*feel more normal*.”^43^ They enjoyed the ability to play more sports—“*I can do the things I like the best, and [it] is just fantastic*.”^65^ Others could travel spontaneously and they “*[were] able to take a vacation*.”^56^ Participants felt more productive and worked more “*effectively, as [they] no longer need[ed] to keep stopping and checking blood glucose via blood tests*.”^58^

## 7 DEVELOPING CONFIDENCE

### 7.1 Encouraged by the endorsement of others

Endorsement of the use of CGM from physicians and nurses increased patients' confidence in the CGM/insulin pump therapy's ability to improve their diabetes to get “*best possible treatments*.”^33^ Participants trusted their clinicians to be knowledgeable and were interested to “*use this machine because [their] attending physician introduced it to [them]*.”^30^ Participants were able to derive more benefits from CGM when clinicians provided them with “*support*”^35^ and encouragement—“*They looked at the graph and then just said: ‘You're doing a great job’*.”^60^ Participants were encouraged to use CGM when they get “*active familiar support*.”^48^

### 7.2 Gaining operational skills

Routine appointments with the diabetes team that included CGM training was “*more informative*”^30^ on “*what's normal and what's not normal*”,^67^ including the ability to understand when “*turning off [alert] what's not useful*.”^68^

### 7.3 Customizing settings for ease of use

Some reported that CGM was “*super easy*”^67^ and “*pretty simplistic*”^63^ to use. Participants liked the simple and “*aesthetic*”^40^ screen layout with the ability to customize settings allowed for individualized diet and exercise inputs.

### 7.4 Trust in the device

Patients were “*confident in what [their CGM] says*,”^63^ and felt reassured when they gained confidence in the accuracy of the device. Trust in the device allowed participants to feel secure enough on their diabetes control to CGM – “*10% of the time it's wrong or not reading correctly*. *I'm clearly going to keep wearing it and trusting it for the other 90% of the time*.”^68^

## 8 BURDEN WITH DEVICE COMPLEXITIES

### 8.1 Bewildered by unfamiliar technology

Older patients found it difficult to operate CGM and felt that learning new technology was “*too troublesome*.”^30^ Adjustment to the new technology was stressful and there was fear surrounding the uncertainty of what to expect and the need to “*be mentally prepared for different situations that can arise [from using CGM]*.”^35^ A lack of familiarity and understanding of CGM/insulin pump therapy contributed to the anxiety relating to its use and the concern that they “*would not understand what [the device] is*.”^60^

### 8.2 Reluctant to rely on algorithms

Participants were skeptical and suspicious of using the machines and having to rely on automated processes to manage their disease. Some felt the attachment to the CGM was akin to being “*kind of like a machine*.”^62^ Participants were also reluctant to embrace a more passive approach to their diabetes—“*When I would eat something or do something, I would get up and get my insulin but now on the pump, I don't really have to do that so I'm a bit lazier*.”^35^

### 8.3 Overwhelmed by data

Some reported that the input and calibration required in using the CGM device was “*too demanding*,”^65^ and felt the process was tedious and relentless, and “*there are more things where you must think more, where before it was just a pen*.”^33^ CGM generated large amounts of data such that participants were unable to “*decipher what's useful and what can sort of be left out*.”^62^ Participants felt demotivated by poor glucose readings as it was “*frustrating [to learn of] fluctuations you previously were not aware*.”^74^

### 8.4 Frustrated with malfunctioning and inaccuracy

Patients with type 1 diabetes felt frustrated with technical failure and sensor problems—“*I had to touch the screen three to five times before it would register the contact*.”^66^ Participants were concerned that the sensors could become dislodged as they were uncomfortable to wear and did not adhere properly. Some reported that the “*discrepancy between blood and sensor glucose [was] confusing*.”^56^ The accuracy and reliability of CGM were felt to be “*still a little bit flaky*”^35^ because “*[the device] gives a false sense of security that [patients] only realize when [they]’ve been disappointed*,”^54^ and some felt the technology should be improved.

### 8.5 Distressed by alerts

Participants with type 1 diabetes reported that frequent alarms were distressing and intrusive for patients who felt they were “*living by alarms*,”^58^ which consequently impaired their mental and physical health. Their frustration was exacerbated by false alarms or if they were uncertain as to how to turn off the alarm. At times, participants could not differentiate between genuine alarms that warranted corrective glycemic action, and alarms that could be ignored, because they did not “*know why [the CGM was] doing what it's doing*.”^61^

### 8.6 Bulkiness of machines interfering with lifestyle

Participants reported that the bulkiness of machines was burdensome to carry, and there were worries “*of breaking things*”^32^ if handled inappropriately. Participants felt uncomfortable and embarrassed with having a device permanently attached to the body, and the “*visibility of the device*”^56^ prevented concealing the disease from people who “*ask questions*.”^43^ One participant noted: “*In the summer, adhesive doesn't last long, so I need to take care of extra fixing, especially if I'm going to the beach*.”^54^ Some were bothered that the CGM device “*pulled skin when [they] moved*,”^34^ and caused “*inconvenience while being intimate with their partner*.”^74^

## 9 EXCLUDED BY BARRIERS TO ACCESS

### 9.1 Constrained by cost

Younger participants were concerned about the cost for CGM/insulin pump therapy—“*Over 50 dollars apiece and if you screw it up, you throw out money*.”^35^ Some felt “*frustrated that the [health system] won't fund [the device]*,”^58^ and remarked: “*I want an artificial pancreas, but I don't want to be broke like I can't go out with my mates*. *I don't want to stop worrying about my diabetes but then have to worry about money*.”^22^ Even in countries with subsidy for CGM, there were concerns about the sustainability of funding to support long‐term use, and patients “*worry that [they] may have to stop using CGM when finances get tight*.”^58^

### 9.2 Lack of supplies

Some were discouraged because there were limited suppliers of the machine, with long waiting times and having to navigate a “*bureaucracy nightmare*”^75^ to access CGM. Participants from rural areas delays in obtaining the device—“*It could easily take four days before I got to the hospital to get new [sensors]*”^65^ and highlighted inequities of accessing CGM.

## 10 DISCUSSION

For people with diabetes, CGM reduced pain associated to glucometer testing and overall treatment burden in the management of diabetes compared to standard monitoring of blood glucose. The use of CGM empowered patients in preventing hypoglycemic crises, motivated them in achieving glucose targets with reduced HbA1c, and enabled them to share responsibility of their care with family members and friends. Increased confidence and improved understanding of lifestyle and glucose interaction encouraged patients with diabetes to engage with their glycemic readings and allowed them to take ownership of their condition. The automation of CGM provided patients with diabetes to have a peace of mind and gave them freedom for social participation. Endorsement from health professionals or caregivers helped patients in gaining operational skills, customizing settings, and trusting the device. On the other hand, some patients reported stressful and frustrating encounters with algorithms and malfunctioning of the device and felt overwhelmed by the amount of data. Some felt distressed by the frequency of alarms and inconvenience with the bulky device causing constraints or embarrassment from visibility. They were also concerned that CGM was unaffordable or there was lack of supplies that prevented uptake of the device.

Although many of the themes were similar across the studies conducted in different populations and settings, there were apparent differences in perspectives based on age, type of diabetes, type of CGM used, and resource settings. Older patients raised concerns around operating the device and were unfamiliar with technological aspects. Patients with type 1 diabetes were less concerned with risks of hypoglycemia since using the device but reported more distress with alerts and malfunctions than patients with type 2 diabetes. Patients with type 1 diabetes were more motivated to set goals for their treatment to prevent diabetic complications, whereas patients with type 2 diabetes paid more attention to their condition since using CGM. Patients reported that CGM supported motivation for diet and lifestyle changes, which resulted in better glycemic control. Patients who used CGM only without an augmented insulin pump reported attaining better insight into the cause of the effect of their lifestyle, insulin treatment, and blood sugar and were more motivated to engage in behavioral changes. Instead, patients using CGM augmented insulin pumps reported an emphasis on developing trust in the automated process and increased feelings of safety due to the accuracy of the readings. Some participants in low resource settings could not afford CGM due to the financial burden or lack of supplies.

Previous systematic reviews have found that CGM was easy to use and improved patient empowerment and autonomy in management of glucose levels, preventing hypoglycemia.^78^, ^79^ CGM minimized burden of diabetes, including regime‐related and interpersonal distress, and reduced pain and discomfort when using the device.^80^, ^81^, ^82^ CGM data sharing enabled family to better manage patient's glucose and patients felt more supported during their journey.^78^ Similarly, studies performed in people using an insertable cardiac device, including pacemakers, felt motivated to make adjustments based on health information recorded in the device and reassured in having their condition under control.^83^, ^84^ However, patients with type 1 and type 2 diabetes have reported technical difficulties associated with CGM use, such as navigation of the CGM menu, management of calibration or sensor errors, discomfort in wearing the sensor, and frustration by responding to alerts.^78^, ^79^ Patients reported mistrust in accuracy of automation and algorithms provided by CGM^78^ or lack of reimbursement that affected their adherence.^85^ This study emphasizes that CGM was less painful and time consuming compared to standard monitoring of blood glucose, increasing patient awareness in interpreting results and promoting lifestyle changes to attain HbA1c targets. CGM provided patients with reassurance that their glucose levels were under control and suggested that health professionals should educate and train patients to increase their operational skills and trust in the device. Of note, our synthesis highlighted that patients felt overwhelmed by the amount of data, and reported inequity in accessing the device, particularly in low resources settings.

In our systematic review, we conducted a comprehensive search, assessed the transparency of study reporting, and used an explicit framework to assess and synthesize the findings. We used investigator triangulation to ensure that we captured the breadth and depth data across the included studies. However, our study has some potential limitations. Some clinical information, including the patient's type of diabetes or the type of insulin regime, were not reported in the primary studies. It was difficult to identify the type and model of CGM used, if the CGM augmented insulin pump included either an open loop or closed loop device, provide a clear difference between flash CGM to standard CGM, or identify the “generation” of CGM used where modern CGM may be nonadjunctive, comparatively slim fitting, more accurate, and often calibration free as these data were not explicitly reported in the included studies. As such, we could not evaluate possible differences based on these characteristics. We included studies published in English only, and most studies were conducted in high‐income countries, which may limit the transferability of our findings.

This study identified potential areas of relevance to clinical practice. Participants identified that CGM was valuable in preventing adverse events and improving lifestyle changes to adhere with glucose targets. However, patients identified limitations in their understanding, the need for technical support, customized device settings, and ergonomic improvements. We suggest that clinicians provide ongoing diabetes education and training on how to interpret the results and technical operations, involving family members, as needed.^86^ Social media or support groups may help patients with diabetes to get practical tips on the management and insertion of the device from people experiencing the same condition. Since the COVID‐19 pandemic, CGM and sensor‐augmented insulin pump therapy may be a helpful tool for sending results to health professionals for review and discussion in a consistent manner and inform treatment strategies for managing diabetes.^87^, ^88^ However, digital technologies suppliers should ensure that CGM devices are optimized, particularly for frail or dependent patients to increase the uptake in these populations, and improved to reduce visibility and bulkiness. We also advocate for financial assistance and subsidies for patients in underprivileged countries to access CGM and sensor‐augmented insulin pump therapy, to decrease long‐term costs for both the health care system and patients.^89^

Self‐management of diabetes has been identified as a high‐priority research question by patients and health professionals.^90^ Further research on interventions to improve the uptake of CGM and sensor‐augmented insulin pump therapy should address patient perspectives and include outcomes of importance to patients to guide their implementation in clinical practice. Based on the INVOLVE^91^ and Patient‐Centered Outcomes Research Institute (PCORI)^92^ initiatives that promote patient involvement in research, we recommend that patients should be involved in both developing and evaluating interventions to strengthen uptake. Studies of diabetes often evaluate surrogate outcomes (eg, HbA1c), whereas mortality, cardiovascular events, amputation, or patient‐reported outcomes (eg, health‐related quality of life) have been inconsistently reported,^93^ limiting our ability to explore the effects of CGM on outcomes that are meaningful to patients.

CGM and sensor‐augmented insulin pump therapy can increase motivation for self‐management, glycemic control, and confidence in treatments. However, the challenges of uncertainty in readings, technical complexities, bulkiness and visibility of the device, access barriers, and cost remain. A knowledgeable and supportive treating team may help to reduce the practical and psychological burden in patients with type 1 and type 2 diabetes and achieve better patient outcomes.

## AUTHOR CONTRIBUTIONS

Research idea and study design: all authors; data acquisition: Patrizia Natale, Sharon Chen, Ayano Kelly; data analysis/interpretation: all authors; supervision or mentorship: Allison Jaure, Jonathan C. Craig. Each author contributed important intellectual content during manuscript drafting or revision and accepts accountability for the overall work by ensuring that questions pertaining to the accuracy or integrity of any portion of the work are appropriately investigated and resolved. Patrizia Natale is the guarantor of this work and, as such, had full access to all the data in the study and takes responsibility for the integrity of the data and the accuracy of the data analysis.

## CONFLICT OF INTEREST STATEMENT

All authors have no conflicts of interest.

## Supporting information


**Data S1.** Supporting Information
