# Supplementary material for: Patient experiences of continuous glucose monitoring and sensor‐augmented insulin pump therapy for diabetes: A systematic review of qualitative studies
Source: J Diabetes. 2023 Aug 8;15(12):1048–69. doi: 10.1111/1753-0407.13454 (PMC10755613; doi:10.1111/1753-0407.13454)
Supplement: Supplementary file 1 — Data S1. Supporting Information [file JDB-15-1048-s001.docx]

**Supplementary Materials**

**Table S1: Search strategies**

**MEDLINE 1946 to April 19, 2023**

1. qualitative$.tw.

2. exp qualitative research/

3. interview$.tw.

4. focus group$.tw.

5. (thematic$ or theme$).tw.

6. ethnograph$.tw.

7. phenomenon$.tw.

8. symbolic interact$.tw.

9. grounded theory.tw.

10. quality of life/

11. psychology, social/

12. anxiety/ or depression/

13. emotions/

14. satisfaction/

15. process evaluation$.tw.

16. exp Health Behavior/ or exp Health/

17. exp patients/

18. patient$.tw.

19. exp Chronic Disease/

20. chronic$.tw.

21. Exp diabetes mellitus/

22. Diabetes$.tw

23. exp biosensing techniques/

24. (sensor or sensors or sensing$).tw.

25. (biosensor$ or biosensing$).tw.

26. exp Wearable Electronic Devices/

27. wearable$.tw.

28. Continuous glucose monitor$.tw

29. Insulin pump$.tw

30. or/1-16

31. or/17-22

32. or/23-29

33. 30 and 31 and 32

**Embase 1974 to April 19, 2023**

1. exp biosensor/

2. (sensor or sensors or sensing$).tw.

3. (biosensor$ or biosensing$).tw.

4. exp sensor/

5. wearable$.tw.

6. Continuous glucose monitor$.tw

7. Insulin pump$.tw

8. qualitative$.tw.

9. exp qualitative research/

10. interview$.tw.

11. focus group$.tw.

12. (thematic$ or theme$).tw.

13. ethnograph$.tw.

14. phenomenon$.tw.

15. symbolic interact$.tw.

16. grounded theory.tw.

17. quality of life/

18. exp social psychology/

19. exp satisfaction/

20. exp patients/

21. patient$.tw.

22. exp chronic disease/

23. chronic$.tw.

24. Exp diabetes mellitus/

25. Diabetes$.tw

26. or/1-7

27. or/8-19

28. or/20-25

29. 26 and 27 and 28

**PsycINFO 1806 to April 19, 2023**

1. (sensor or sensors or sensing$).tw.

2. (biosensor$ or biosensing$).tw.

3. wearable$.tw.

4. Glucose monitor$.tw

5. Insulin pump$.tw

6. Technology$.tw

7. qualitative$.tw.

8. exp Qualitative Methods/

9. interview$.tw.

10. focus group$.tw.

11. (thematic$ or theme$).tw.

12. ethnograph$.tw.

13. phenomenon$.tw.

14. symbolic interact$.tw.

15. grounded theory.tw.

16. quality of life/

17. exp Social Psychology/

18. exp Emotions/

19. exp Life Experiences/

20. exp Patients/ or exp Medical Patients/

21. patient$.tw.

22. exp "Chronicity (Disorders)"/ or exp Health Behavior/ or exp Chronic Illness/ or exp Self-Management/ or exp Disease Management/

23. chronic$.tw.

24. Diabetes mellitus/

25. Diabetes$.tw

26. or/1-6

27. or/7-19

28. or/20-25

29. 26 and 27 and 28

**CINAHL to April 19, 2023**

S1. (MH “Wearable Sensors”)

S2. (MH "Blood Glucose Self-Monitoring") OR (MH "Blood Glucose Monitoring+")

S3. (MH "Insulin Infusion Systems")

S4. (MH "Diabetes Mellitus+")

S5. (MH “Qualitative Studies+”)

S6. S2 OR S3 OR S4

S7. S1 AND S6

S8. S5 AND S7

S.9 S5 AND S7

**Table S2. COREQ appraisal**

| **Item** | **Addala 2021** | **Adu 2019** | **Agarwal 2021** | **Allen 2021** | **Barnard 2017** | **Bispham 2021** | **Boucher 2019** | **Burniside 2023** | **Chang 2017** | **Chesser 2022** | **Chiu 2019** | **Cleal 2021** | **Cuervas 2022** |
| --- | --- | --- | --- | --- | --- | --- | --- | --- | --- | --- | --- | --- | --- |
| Personal Characteristics |  |  |  |  |  |  |  |  |  |  |  |  |  |
| Interviewer/facilitator identified | 0 | 1 | 0 | 1 | 1 | 1 | 1 | 1 | 0 | 1 | 0 | 0 | 1 |
| Occupation of the interview/facilitator | 0 | 1 | 0 | 1 | 0 | 1 | 1 | 1 | 0 | 0 | 0 | 0 | 1 |
| Experience or training in qualitative research | 0 | 1 | 0 | 1 | 0 | 0 | 1 | 1 | 1 | 0 | 1 | 0 | 0 |
| Relationship with participants |  |  |  |  |  |  |  |  |  |  |  |  |  |
| Relationship established before study start | 0 | 1 | 0 | 1 | 1 | 0 | 1 | 1 | 0 | 0 | 0 | 0 | 0 |
| Participant selection |  |  |  |  |  |  |  |  |  |  |  |  |  |
| Selection strategy | 0 | 1 | 0 | 0 | 1 | 0 | 1 | 0 | 1 | 1 | 1 | 1 | 1 |
| Method of approach or recruitment | 0 | 1 | 0 | 0 | 1 | 1 | 1 | 1 | 0 | 1 | 0 | 0 | 1 |
| Sample size | 1 | 1 | 1 | 1 | 1 | 1 | 1 | 1 | 1 | 0 | 1 | 1 | 1 |
| No. and/or reasons for nonparticipation | 0 | 1 | 1 | 0 | 0 | 0 | 1 | 1 | 0 | 1 | 0 | 0 | 0 |
| Setting |  |  |  |  |  |  |  |  |  |  |  |  |  |
| Venue of data collection | 0 | 1 | 1 | 0 | 0 | 1 | 1 | 1 | 1 | 1 | 1 | 1 | 1 |
| Presence of nonparticipants (e.g. clinical staff) | 0 | 1 | 0 | 0 | 0 | 0 | 0 | 1 | 0 | 0 | 0 | 0 | 0 |
| Description of sample | 1 | 1 | 1 | 1 | 1 | 1 | 1 | 1 | 1 | 1 | 1 | 1 | 1 |
| Data collection |  |  |  |  |  |  |  |  |  |  |  |  |  |
| Questions, prompts or topic guide | 0 | 1 | 1 | 1 | 1 | 1 | 1 | 1 | 1 | 1 | 1 | 1 | 1 |
| Repeat interviews/observations | 0 | 1 | 0 | 0 | 1 | 0 | 0 | 0 | 0 | 0 | 1 | 0 | 0 |
| Audio/visual recording | 0 | 1 | 1 | 1 | 1 | 1 | 1 | 1 | 1 | 0 | 1 | 0 | 1 |
| Field notes | 0 | 1 | 1 | 0 | 0 | 0 | 0 | 0 | 1 | 0 | 0 | 0 | 0 |
| Duration of data collection | 1 | 1 | 0 | 0 | 1 | 0 | 1 | 1 | 1 | 1 | 1 | 1 | 1 |
| Translation and interpretation | 0 | N/A | 0 | N/A | 0 | N/A | N/A | 0 | 0 | N/A | 0 | 0 | N/A |
| Data preparation and transcription | 0 | 1 | 1 | 1 | 1 | 1 | 1 | 1 | 1 | 0 | 1 | 1 | 0 |
| Data (or theoretical) saturation | 1 | 1 | 1 | 0 | 1 | 0 | 1 | 1 | 0 | 0 | 1 | 0 | 0 |
| Data Analysis |  |  |  |  |  |  |  |  |  |  |  |  |  |
| Researcher/expert triangulation | 1 | 1 | 1 | 1 | 1 | 1 | 1 | 1 | 1 | 1 | 0 | 0 | 1 |
| Translation | 0 | N/A | 0 | N/A | 0 | N/A | N/A | 0 | 0 | N/A | 0 | 0 | N/A |
| Derivation of themes or findings | 1 | 1 | 1 | 1 | 1 | 1 | 1 | 1 | 1 | 1 | 1 | 1 | 1 |
| Use of software | 1 | 1 | 1 | 1 | 0 | 0 | 1 | 1 | 1 | 0 | 0 | 1 | 1 |
| Member checking | 0 | 0 | 0 | 0 | 0 | 0 | 0 | 1 | 0 | 1 | 0 | 0 | 0 |
| Reporting |  |  |  |  |  |  |  |  |  |  |  |  |  |
| Participant quotations or raw data provided | 1 | 1 | 1 | 1 | 1 | 1 | 1 | 1 | 1 | 1 | 1 | 1 | 1 |
| Range of depth of insight into participant perspectives using CGM | 0 | 1 | 1 | 1 | 0 | 0 | 1 | 0 | 1 | 0 | 1 | 1 | 1 |

| **Item** | **Dutil 2014** | **Farrington 2018** | **Faulds 2022** | **Fritschi 2022** | **Gajewska 2021** | **Grando 2019*** | **Griauzde 2022** | **Grigorian 2022** | **Haynes 2021** | **Hendrieckx 2017** | **Hughes 2022** | **Kahkoska 2023** |
| --- | --- | --- | --- | --- | --- | --- | --- | --- | --- | --- | --- | --- |
| Personal Characteristics |  |  |  |  |  |  |  |  |  |  |  |  |
| Interviewer/facilitator identified | 0 | 0 | 1 | 1 | 1 | N/A | 1 | 1 | 1 | 1 | 1 | 0 |
| Occupation of the interview/facilitator | 0 | 0 | 0 | 1 | 0 | N/A | 1 | 1 | 1 | 1 | 1 | 1 |
| Experience or training in qualitative research | 0 | 0 | 0 | 1 | 0 | N/A | 1 | 1 | 1 | 1 | 0 | 0 |
| Relationship with participants |  |  |  |  |  |  |  |  |  |  |  |  |
| Relationship established before study start | 0 | 0 | 0 | 1 | 0 | N/A | 1 | 1 | 0 | 1 | 0 | 0 |
| Participant selection |  |  |  |  |  |  |  |  |  |  |  |  |
| Selection strategy | 0 | 1 | 1 | 1 | 1 | N/A | 1 | 0 | 1 | 0 | 0 | 1 |
| Method of approach or recruitment | 1 | 0 | 0 | 1 | 1 | N/A | 1 | 1 | 1 | 0 | 1 | 1 |
| Sample size | 1 | 1 | 1 | 1 | 1 | N/A | 1 | 1 | 1 | 1 | 1 | 1 |
| No. and/or reasons for nonparticipation | 0 | 1 | 0 | 0 | 0 | N/A | 1 | 0 | 1 | 0 | 0 | 0 |
| Setting |  |  |  |  |  |  |  |  |  |  |  |  |
| Venue of data collection | 0 | 0 | 1 | 1 | 0 | N/A | 1 | 1 | 1 | 1 | 1 | 1 |
| Presence of nonparticipants (e.g. clinical staff) | 0 | 0 | 0 | 0 | 0 | N/A | 0 | 0 | 0 | 0 | 0 | 1 |
| Description of sample | 1 | 1 | 1 | 1 | 1 | N/A | 1 | 1 | 1 | 1 | 1 | 1 |
| Data collection |  |  |  |  |  |  |  |  |  |  |  |  |
| Questions, prompts or topic guide | 1 | 1 | 0 | 1 | 1 | N/A | 0 | 1 | 1 | 1 | 1 | 1 |
| Repeat interviews/observations | 0 | 1 | 0 | 0 | 0 | N/A | 0 | 1 | 1 | 0 | 0 | 0 |
| Audio/visual recording | 1 | 1 | 1 | 1 | 1 | N/A | 1 | 1 | 1 | 1 | 1 | 1 |
| Field notes | 1 | 0 | 1 | 0 | 0 | N/A | 0 | 0 | 0 | 0 | 1 | 0 |
| Duration of data collection | 0 | 0 | 0 | 1 | 1 | N/A | 0 | 1 | 1 | 1 | 1 | 1 |
| Translation and interpretation | N/A | N/A | N/A | 0 | 0 | N/A | N/A | N/A | 0 | N/A | N/A | N/A |
| Data preparation and transcription | 1 | 1 | 0 | 0 | 1 | N/A | 0 | 0 | 1 | 1 | 1 | 0 |
| Data (or theoretical) saturation | 1 | 1 | 0 | 0 | 0 | N/A | 1 | 0 | 1 | 0 | 0 | 1 |
| Data Analysis |  |  |  |  |  |  |  |  |  |  |  |  |
| Researcher/expert triangulation | 1 | 0 | 1 | 1 | 1 | N/A | 1 | 1 | 1 | 1 | 1 | 1 |
| Translation | N/A | N/A | N/A | 0 | 0 | N/A | N/A | N/A | 0 | N/A | N/A | N/A |
| Derivation of themes or findings | 1 | 1 | 1 | 1 | 1 | N/A | 1 | 1 | 1 | 1 | 1 | 0 |
| Use of software | 0 | 1 | 1 | 0 | 1 | N/A | 1 | 1 | 1 | 1 | 0 | 1 |
| Member checking | 0 | 0 | 0 | 1 | 0 | N/A | 1 | 1 | 1 | 0 | 0 | 1 |
| Reporting |  |  |  |  |  |  |  |  |  |  |  |  |
| Participant quotations or raw data provided | 1 | 1 | 0 | 1 | 1 | N/A | 1 | 1 | 1 | 1 | 1 | 1 |
| Range of depth of insight into participant perspectives using CGM | 1 | 0 | 0 | 1 | 1 | N/A | 0 | 0 | 1 | 0 | 0 | 1 |

| **Item** | **Kang 2022** | **Kaisen 20120** | **Kropff 2017** | **Litchman 2018*** | **Litchman 2021** | **Lukacs 2022*** | **Nadeem 2021*** | **Overend 2019** | **Persson 2022** | **Pickup 2015*** |
| --- | --- | --- | --- | --- | --- | --- | --- | --- | --- | --- |
| Personal Characteristics |  |  |  |  |  |  |  |  |  |  |
| Interviewer/facilitator identified | 0 | 1 | 0 | N/A | 0 | N/A | N/A | 1 | 1 | N/A |
| Occupation of the interview/facilitator | 0 | 1 | 0 | N/A | 0 | N/A | N/A | 1 | 1 | N/A |
| Experience or training in qualitative research | 0 | 1 | 0 | N/A | 0 | N/A | N/A | 0 | 0 | N/A |
| Relationship with participants |  |  |  |  |  |  |  |  |  |  |
| Relationship established before study start | 1 | 0 | 0 | N/A | 1 | N/A | N/A | 1 | 1 | N/A |
| Participant selection |  |  |  |  |  |  |  |  |  |  |
| Selection strategy | 1 | 0 | 0 | N/A | 1 | N/A | N/A | 1 | 0 | N/A |
| Method of approach or recruitment | 1 | 1 | 0 | N/A | 1 | N/A | N/A | 1 | 1 | N/A |
| Sample size | 1 | 1 | 1 | N/A | 1 | N/A | N/A | 1 | 1 | N/A |
| No. and/or reasons for nonparticipation | 0 | 0 | 0 | N/A | 1 | N/A | N/A | 0 | 1 | N/A |
| Setting |  |  |  |  |  |  |  |  |  |  |
| Venue of data collection | 1 | 1 | 0 | N/A | 1 | N/A | N/A | 1 | 1 | N/A |
| Presence of nonparticipants (e.g. clinical staff) | 0 | 0 | 0 | N/A | 0 | N/A | N/A | 0 | 0 | N/A |
| Description of sample | 1 | 1 | 0 | N/A | 0 | N/A | N/A | 1 | 1 | N/A |
| Data collection |  |  |  |  |  |  |  |  |  |  |
| Questions, prompts or topic guide | 1 | 1 | 1 | N/A | 0 | N/A | N/A | 1 | 0 | N/A |
| Repeat interviews/observations | 0 | 0 | 0 | N/A | 0 | N/A | N/A | 1 | 0 | N/A |
| Audio/visual recording | 1 | 1 | 0 | N/A | 1 | N/A | N/A | 0 | 1 | N/A |
| Field notes | 0 | 1 | 0 | N/A | 0 | N/A | N/A | 0 | 0 | N/A |
| Duration of data collection | 1 | 0 | 0 | N/A | 0 | N/A | N/A | 0 | 1 | N/A |
| Translation and interpretation | N/A | N/A | 1 | N/A | 1 | N/A | N/A | N/A | N/A | N/A |
| Data preparation and transcription | 1 | 1 | 0 | N/A | 0 | N/A | N/A | 1 | 1 | N/A |
| Data (or theoretical) saturation | 1 | 1 | 1 | N/A | 0 | N/A | N/A | 0 | 0 | N/A |
| Data Analysis |  |  |  |  |  |  |  |  |  |  |
| Researcher/expert triangulation | 1 | 1 | 0 | N/A | 0 | N/A | N/A | 1 | 1 | N/A |
| Translation | N/A | N/A | 1 | N/A | 1 | N/A | N/A | N/A | N/A | N/A |
| Derivation of themes or findings | 1 | 1 | 0 | N/A | 1 | N/A | N/A | 1 | 1 | N/A |
| Use of software | 1 | 0 | 0 | N/A | 0 | N/A | N/A | 0 | 1 | N/A |
| Member checking | 0 | 0 | 0 | N/A | 0 | N/A | N/A | 0 | 1 | N/A |
| Reporting |  |  |  |  |  |  |  |  |  |  |
| Participant quotations or raw data provided | 1 | 1 | 1 | N/A | 0 | N/A | N/A | 1 | 1 | N/A |
| Range of depth of insight into participant perspectives using CGM | 1 | 1 | 0 | N/A | 0 | N/A | N/A | 1 | 1 | N/A |

| **Item** | **Pillamarri 2018*** | **Ritholz 2019** | **Ritholz 2014** | **Ritholz 2010** | **Sawyer 2021** | **Shephard 2012** | **Sørgård2019** | **Stuckey 2021*** | **Tanenbaum 2021a** | **Tanenbau 2021b** |
| --- | --- | --- | --- | --- | --- | --- | --- | --- | --- | --- |
| Personal Characteristics |  |  |  |  |  |  |  |  |  |  |
| Interviewer/facilitator identified | N/A | 1 | 0 | 1 | 1 | 1 | 1 | N/A | 1 | 0 |
| Occupation of the interview/facilitator | N/A | 1 | 0 | 1 | 1 | 0 | 1 | N/A | 1 | 0 |
| Experience or training in qualitative research | N/A | 1 | 1 | 0 | 0 | 0 | 0 | N/A | 1 | 0 |
| Relationship with participants |  |  |  |  |  |  |  |  |  |  |
| Relationship established before study start | N/A | 0 | 0 | 0 | 1 | 0 | 1 | N/A | 1 | 1 |
| Participant selection |  |  |  |  |  |  |  |  |  |  |
| Selection strategy | N/A | 1 | 1 | 1 | 1 | 0 | 1 | N/A | 1 | 1 |
| Method of approach or recruitment | N/A | 1 | 1 | 1 | 1 | 1 | 1 | N/A | 1 | 1 |
| Sample size | N/A | 1 | 1 | 1 | 1 | 1 | 1 | N/A | 1 | 1 |
| No. and/or reasons for nonparticipation | N/A | 1 | 1 | 1 | 0 | 1 | 1 | N/A | 0 | 1 |
| Setting |  |  |  |  |  |  |  |  |  |  |
| Venue of data collection | N/A | 1 | 0 | 0 | 1 | 0 | 1 | N/A | 1 | 0 |
| Presence of nonparticipants (e.g. clinical staff) | N/A | 0 | 0 | 0 | 0 | 0 | 0 | N/A | 0 | 0 |
| Description of sample | N/A | 1 | 1 | 1 | 1 | 1 | 1 | N/A | 1 | 1 |
| Data collection |  |  |  |  |  |  |  |  |  |  |
| Questions, prompts or topic guide | N/A | 1 | 1 | 1 | 1 | 1 | 1 | N/A | 1 | 0 |
| Repeat interviews/observations | N/A | 0 | 0 | 0 | 0 | 0 | 1 | N/A | 0 | 0 |
| Audio/visual recording | N/A | 1 | 1 | 1 | 1 | 0 | 1 | N/A | 1 | 1 |
| Field notes | N/A | 0 | 1 | 0 | 1 | 0 | 1 | N/A | 0 | 0 |
| Duration of data collection | N/A | 1 | 1 | 1 | 1 | 1 | 1 | N/A | 1 | 0 |
| Translation and interpretation | N/A | N/A | N/A | N/A | N/A | N/A | 0 | N/A | 0 | 0 |
| Data preparation and transcription | N/A | 1 | 1 | 1 | 1 | 1 | 1 | N/A | 0 | 0 |
| Data (or theoretical) saturation | N/A | 1 | 1 | 1 | 0 | 0 | 0 | N/A | 1 | 0 |
| Data Analysis |  |  |  |  |  |  |  |  |  |  |
| Researcher/expert triangulation | N/A | 1 | 1 | 1 | 0 | 0 | 1 | N/A | 1 | 1 |
| Translation | N/A | N/A | N/A | N/A | N/A | N/A | 1 | N/A | 0 | 0 |
| Derivation of themes or findings | N/A | 1 | 1 | 1 | 1 | 1 | 1 | N/A | 1 | 1 |
| Use of software | N/A | 1 | 1 | 0 | 0 | 0 | 1 | N/A | 1 | 0 |
| Member checking | N/A | 0 | 0 | 1 | 0 | 0 | 1 | N/A | 1 | 0 |
| Reporting |  |  |  |  |  |  |  |  |  |  |
| Participant quotations or raw data provided | N/A | 1 | 1 | 1 | 1 | 0 | 1 | N/A | 1 | 1 |
| Range of depth of insight into participant perspectives using CGM | N/A | 1 | 1 | 1 | 1 | 0 | 1 | N/A | 1 | 0 |

| **Item** | **Toschi 2022** | **Tzivian 2022** | **Vallis 2022** | **Van Heerden 2022** | **Vlcek 2023** | **Vloemans 2017** | **Walker 2021** | **Wallace 2023** | **Yorgason 2021** |
| --- | --- | --- | --- | --- | --- | --- | --- | --- | --- |
| Personal Characteristics |  |  |  |  |  |  |  |  |  |
| Interviewer/facilitator identified | 0 | 0 | 1 | 1 | 1 | 1 | 0 | 1 | 1 |
| Occupation of the interview/facilitator | 0 | 0 | 1 | 1 | 1 | 1 | 1 | 1 | 1 |
| Experience or training in qualitative research | 0 | 0 | 1 | 1 | 1 | 0 | 1 | 0 | 1 |
| Relationship with participants |  |  |  |  |  |  |  |  |  |
| Relationship established before study start | 0 | 1 | 1 | 0 | 0 | 0 | 0 | 1 | 0 |
| Participant selection |  |  |  |  |  |  |  |  |  |
| Selection strategy | 0 | 0 | 1 | 1 | 1 | 1 | 0 | 0 | 1 |
| Method of approach or recruitment | 0 | 1 | 1 | 0 | 1 | 0 | 1 | 1 | 0 |
| Sample size | 1 | 1 | 1 | 1 | 1 | 1 | 1 | 1 | 1 |
| No. and/or reasons for nonparticipation | 0 | 1 | 1 | 0 | 0 | 1 | 0 | 1 | 0 |
| Setting |  |  |  |  |  |  |  |  |  |
| Venue of data collection | 1 | 1 | 1 | 1 | 1 | 0 | 0 | 1 | 0 |
| Presence of nonparticipants (e.g. clinical staff) | 0 | 0 | 0 | 0 | 0 | 0 | 1 | 0 | 0 |
| Description of sample | 1 | 1 | 1 | 1 | 1 | 1 | 1 | 1 | 1 |
| Data collection |  |  |  |  |  |  |  |  |  |
| Questions, prompts or topic guide | 1 | 0 | 1 | 1 | 1 | 1 | 1 | 1 | 1 |
| Repeat interviews/observations | 0 | 0 | 0 | 0 | 0 | 0 | 0 | 0 | 0 |
| Audio/visual recording | 1 | 1 | 1 | 1 | 1 | 1 | 1 | 1 | 1 |
| Field notes | 0 | 0 | 1 | 0 | 0 | 0 | 1 | 0 | 1 |
| Duration of data collection | 1 | 0 | 1 | 1 | 0 | 1 | 1 | 1 | 1 |
| Translation and interpretation | N/A | 1 | 0 | 1 | 1 | N/A | 1 | N/A | N/A |
| Data preparation and transcription | 0 | 0 | 0 | 0 | 1 | 1 | 1 | 1 | 0 |
| Data (or theoretical) saturation | 0 | 1 | 1 | 1 | 1 | 0 | 1 | 0 | 1 |
| Data Analysis |  |  |  |  |  |  |  |  |  |
| Researcher/expert triangulation | 1 | 0 | 1 | 0 | 1 | 1 | 1 | 1 | 1 |
| Translation | N/A | 1 | 0 | 1 | 1 | N/A | 0 | N/A | N/A |
| Derivation of themes or findings | 1 | 1 | 1 | 1 | 1 | 1 | 1 | 1 | 1 |
| Use of software | 1 | 1 | 1 | 1 | 1 | 0 | 0 | 1 | 0 |
| Member checking | 0 | 0 | 1 | 0 | 1 | 0 | 0 | 1 | 1 |
| Reporting |  |  |  |  |  |  |  |  |  |
| Participant quotations or raw data provided | 1 | 1 | 1 | 1 | 1 | 1 | 1 | 1 | 1 |
| Range of depth of insight into participant perspectives using CGM | 1 | 0 | 1 | 1 | 0 | 1 | 1 | 1 | 1 |

* Only interview and focus group studies were included in the COREQ assessment: N/A: Not applicable.
